# Prices paid for primary health care medicines by Brazilian municipalities

**DOI:** 10.11606/s1518-8787.2025059006964

**Published:** 2025-12-08

**Authors:** Wendell Rodrigues Oliveira da Silva, Ivanessa Thaiane do Nascimento Cavalcanti, José Roberto Peters, Luis Eduardo Maciel dos Santos Ferreira, Rafael Santos Santana, Silvana Nair Leite

**Affiliations:** I Universidade de Brasília. Faculdade de Ciências da Saúde, Programa de Pós-graduação em Ciências Farmacêuticas. Brasília, DF, Brazil; II Ministério da Saúde. Departamento de Economia e Desenvolvimento em Saúde. Brasília, DF, Brasil; III Universidade Federal de Santa Catarina. Departamento de Ciências Farmacêuticas, Programa de Pós-graduação em Assistência Farmacêutica. Florianópolis, SC, Brasil

**Keywords:** Pharmaceutical Services, Primary Health Care, Medicines Costs, Equity in Resource Allocation, National Pharmaceutical Policy

## Abstract

**OBJECTIVE:**

The study analyzes the prices paid by Brazilian municipalities for medicines in 2016, 2018, and 2020, comparing municipal human development index, size, region, and purchase modality.

**METHODS:**

Economic Ratio indicator, adapted from that proposed for international analyses by the World Health Organization, was used and analyses were performed using data provided by municipalities to the National Database of Pharmaceutical Assistance Actions and Services.

**RESULTS:**

The study reveals that the most socially and economically vulnerable municipalities pay more for these medicines, especially in the North and Northeast regions, which can compromise access to and the efficiency of the Unified Health System. Moreover, the article shows that municipalities that purchased in association tend to pay lower prices, suggesting that consortia may be a strategy to mitigate price inequalities.

**CONCLUSIONS:**

Based on the results obtained, we propose the adoption of regulatory, economic, and negotiation strategies to reduce these differences and ensure a more equitable distribution of medicines in Brazil.

## INTRODUCTION

High medicines prices worry governments, policymakers, insurers, and patients, as they compromise their equitable access and threaten the sustainability of health systems, regardless of the country’s degree of socioeconomic development^1^.

Medicines prices fluctuate according to the interactions between supply and demand, within the scope of the country’s price regulation^1^. Across countries, factors such as the healthcare system, medicines policy, and free access influence the price and, consequently, the obtaining of medicines^4,5^. Studies on the price, access, and quality of pharmaceutical products are essential in Brazil, since the public supply of medicines is the main way of access for the population that depends on the public healthcare system^5^.

In force for more than 20 years, Law No. 10,742/2003 establishes a price ceiling for medicines in Brazil. Within the pharmaceutical market reality, however, prices can vary by more than 2,500% in the private market^3^. A report by the Federal Court of Auditors identified prices registered in the table of the *Câmara de Regulação do Mercado de Medicamentos* (CMED – Chamber for the Regulation of Medicines Market) above 10,000% of those practiced in public purchases^6^.

**Table:** Comparison of the overall average values of economic ratio in the years analyzed, MHDI, type of purchase, region of the country, and population sizea.

	Mean (95%CI)
Region	
Midwest	1.04 (1.00–1.07)
Northeast	1.13 (1.10–1.16)
North	1.32 (1.28–1.37)
Southeast	0.82 (0.79–0.85)
South	0.78 (0.74–8.82)
Population size	
Up to 25,000 inhabitants	1.02 (1.01–1.03)
25,001 to 50.000 inhabitants	0.98 (0.97–1.00)
50,001 to 100,000 inhabitants	0.93 (0.91–0.95)
100,000 to 500,000 inhabitants	0.93 (0.91–0.95)
Above 500,000 inhabitants	0.88 (0.84–0.92)
MHDI	
Very low	1.37 (1.26–1.47)
Low	1.18 (1.16–1.20)
Medium	0.97 (0.96–0.99)
High	0.85 (0.82–0.87)
Very high	0.75 (0.69–0.80)
Purchases with consortia	
Purchase with consortia	0.82 (0.78–0.85)
Purchase without consortia	1.10 (1.08–1.12)

MHDI: Municipal Human Development Index; 95%CI: 95% confidence interval.

^a^ In this analysis, the values found for the p-value were less than 0.0001, showing that the results are statistically significant to describe the effect of the model.

The offer of medicines depends on health funding, efficient management, availability of products, purchasing power, rationality of use, and geographical accessibility^7^. Public funding and the organization of pharmaceutical services in primary health care (PHC) are fundamental in providing the Brazilian population access to medicines^5,8,9^. In 2015, 47% of the population obtained all their medicines for chronic diseases free of charge from the Brazilian Unified Health System (SUS). However, the Northeast region had the lowest prevalence of access to all medicines^8^.

In the SUS, the purchases of medicines are conducted autonomously by the Union, the states, the Federal District, and the municipalities, with decentralization that enables planning according to local needs. Most PHC medicines are purchased directly by the municipalities, alone or in consortium. The Union invested R$ 132 billion in health and R$ 13 billion in medicines in 2019, while states spent R$ 102 billion and R$ 1.7 billion, respectively. Municipalities invested R$ 5 billion in medicines, according to data from the Information System on Public Health Budgets (SIOPS)^10^.

Ordinance No. 3,193/2019 changed the transfers of the Basic Component of Pharmaceutical Services (CBAF), linking them to the municipal human development index (MHDI). The Union passed on per capita amounts to municipalities annually, varying according to the MHDI classification, data that were updated in 2025^11^. The funding aims at the acquisition of medicines and supplies included in the Brazilian National List of Medicines (Rename) for PHC in the SUS.

Despite decentralization, municipal funding for medicines is worrying, because more vulnerable municipalities invest less and depend more on federal transfers, according to Silva et al.^10^ (2024), a trend observed between 2016 and 2020.

Considering the economic and municipal management conditions and the characteristics of the pharmaceutical market in Brazil, the prices of medicines for PHC can vary greatly between municipalities^12^ and represent an important impact on the municipal capacity to ensure the availability of these medicines, which are essential for the resolvability of PHC and the achievement of better health outcomes.

This study analyzed the prices charged in 2016, 2018, and 2020 in the acquisition, of medicines used in primary health care by Brazilian municipalities, considering their socioeconomic conditions, geographic region, and acquisition modalities.

## METHODS

This is an exploratory, retrospective study, in which the prices of the acquisition of primary health care medicines by the municipalities were analyzed, based on data fed into the *Base Nacional de Dados e Ações e Serviços da Assistência Farmacêutica* (BNAFAR – National Database and Pharmaceutical Services Actions and Services). This integrates and consolidates national data on stock position, entries, exits, and dispensations conducted by health establishments for medicines from Rename and the Popular Pharmacy Program of Brazil, covering the largest number of records of medicines purchases available, especially from municipalities^13^.

To determine the amount paid for the medicines, the recorded values were extracted, in which the most used presentations of the medicines were reference, without discrimination against brands. The data referred to 2016 (2,440 municipalities), 2018 (2,866 municipalities), and 2020 (3,815 municipalities). The choice of these years considered the corrections in the value of the CBAF counterpart by the Ministry of Health in 2017^14^ and in 2019^11^. Data were collected using Business Intelligence (BI) by MicroStrategy Office*.*

The choice of medicines was based on those identified in the *Pesquisa Nacional sobre Acesso, Utilização e Promoção do Uso Racional de Medicamentos* (PNAUM – National Survey on Access, Use, and Promotion of Rational Use of Medicines) as the most used for hypertension, diabetes mellitus, hypercholesterolemia, chronic pulmonary respiratory disease, depression, rheumatism, pain, fever, and infection. For the last three, the authors decided on medicines widely used by the Brazilian population because the PNAUM did not find difference in their use^15^.

Appendix 1^a^ describes medicines selected for analysis. The statistical program Rstudio was used to exclude the *Outliers* of the database, considering the percentage below 1% and above 99% as points outside the curve.

BNAFAR data were extracted from the presentations acquired by the municipalities, with quantities and prices practiced, and the average of the prices paid per unit of supply was calculated.

The inflation adjustment of the amounts paid for the medicines was conducted. These values were deflated for December 2022, according to the annual variation of the Broad Consumer Price Index (IPCA), calculated by the Brazilian Institute of Geography and Statistics – IBGE (2016: 44.32%; 2018: 31.54%; 2020: 21.88%). The choice of this deflator was based on Law No. 10,742/2003, which established the rules for regulating the pharmaceutical sector and defined the index for adjusting the prices of medicines in the country.

The manner in which medicines were purchased by the municipalities was classified as purchase in association or non-associated, distinguishing between purchases among entities (municipality and state), such as intermunicipal consortia, at the regional or state level, and those in which the municipality acquires its medicines in isolation. These data were provided by the General Coordination of Basic Pharmaceutical Services of the Ministry of Health.

Regarding the municipal population size, the stratum model of the Qualifar-SUS Program was used, which stratifies the municipalities: up to 25,000 inhabitants, from 25,001 to 50,000 inhabitants, from 50,001 to 100,000 inhabitants, from 100,001 to 500,000 inhabitants, and municipalities above 500,000 inhabitants^16^.

The municipal human development index (MHDI) was also used. This varies from 0 to 1 (UNDP), and municipalities with an index below 0.499 are considered very low; MHDI is low for municipalities with an index from 0.500 to 0.599; MHDI is medium for municipalities with an index from 0.600 to 0.699; MHDI is high for municipalities with an index from 0.700 to 0.799; and MHDI is very high municipalities with an index equal to or greater than 0.800.

Regional analyses were conducted based on these data, by population size, by MHDI stratum, and by purchases in association or not, to identify and compare the values practiced in the purchase of medicines by Brazilian municipalities.

An adaptation of the “medicines price ratio”, an index developed by the World Health Organization (WHO)/International Health Action, was used^17^. This is a methodological tool for measuring medicines prices (government procurement prices and patient prices), availability, accessibility, and the price components, previously prepared by WHO^18,19^.

In this study, the indicator was adapted to include the information processed in the analyzed sample and to compare the amounts paid, considering the different medicines included in the sample. The “local average unit price” was established as the average of the average price paid for each presentation of medicines by municipality, and as “national reference unit price” the national average price paid for the presentation of medicines that corresponds to the national average of the amounts paid for each presentation of each medicines, considering all the registrations for the purchase of such medicines in BNAFAR, that year. Thus, the adapted Economic Ratio (ER) formula used in this study was:


$$\mathrm{ER}=\frac{\begin{array}{c} \text{ Average of the average price paid for the }\\ \text{ presentation of medicines by the municipality } \end{array}}{\text{ national average price of the presentation of medicines }}$$

As a result, the values obtained for the ER that are equal to one indicate that the amount paid was identical to the national average; for a result lower than one, the price paid was lower than the amount paid for the acquisition of the medicines when compared to the national average; and for a result greater than one, it shows that there was a payment above the national average.

The generalized estimation equation model was used to compare the average of the ER variable among the studied variables. The level of significance adopted was 0.05. The analyses were performed using the IBM SPSS Statistics v.25 software.

## RESULTS

The municipalities in this study are characterized as follows: regarding the geographic region, 351 are from the Midwest region, 1,147 are from Northeast, 256 are from North, 1,310 are from Southeast, and 751 are from the South region. Regarding the population size: 2,894 have up to 25,000 inhabitants, 476 have 25,001 to 50,000 inhabitants, 233 have 50,001 to 100,000 inhabitants, 181 have 100,000 to 500,000 inhabitants, and 31 municipalities have more than 500,000 inhabitants. Regarding the classification of the MHDI, the distribution is as follows: 10 municipalities are classified as very low, 847 as low, 1,680 as medium, 1,250 as high, and 28 as very high MHDI. And regarding the acquisition strategy (with or without association), 1,375 municipalities make associated purchases, 2,391 make purchases without association with other entities and 49 municipalities did not have this information available.

**Figure 1:**
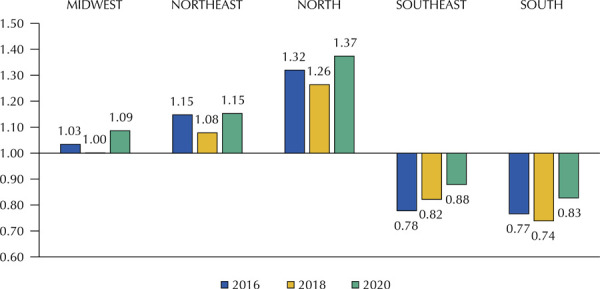
Economic ratio average of the medicines analyzed by region and by year.

Appendix 2^b^ shows in detail the results obtained for prices practiced in the purchases by drug and the corresponding ER by region, size of the municipality, MHDI range, and acquisition modality. Figure 1 shows the average value of the ER of the medicines, regionally, per year. Note that in the three years of the survey the North region had the highest average value of ER.

**Figure 2:**
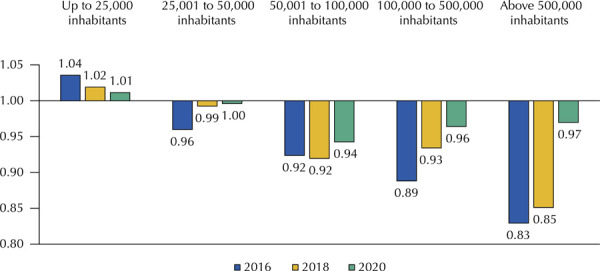
Economic ratio average of the medicines analyzed by population size and by year.

Considering the population size (Figure 2), it can be observed that in 2016, 2018, and 2020, the smaller municipalities, with up to 25,000 inhabitants, had the highest average ER value.

**Figure 3:**
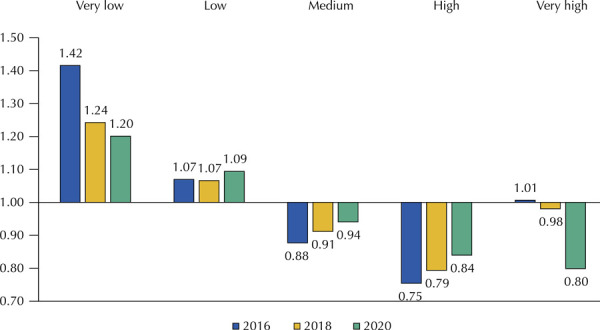
Economic ratio average of the medicines analyzed by MDHI and by year.

In Figure 3, which analyzes the average value of the ER of medicines by MHDI stratum, the municipalities with very low MHDI had the highest average value of ER in the years surveyed.

MHDI: Municipal Human Development Index.

**Figure 4:**
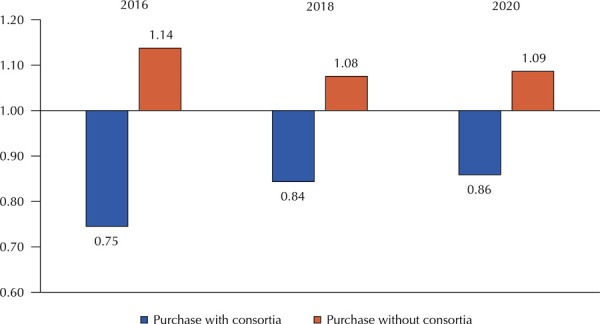
Economic ratio average of the medicines analyzed by purchase in consortia and without consortia, per year.

When analyzing purchases in association or not (Figure 4), the average value of ER in 2016, 2018, and 2020 in the municipalities that did not make the purchase in association was higher in all the years surveyed.

Table shows the comparison of the average values of ER among all the medicines in the variables studied. The North region has the highest general average of ER values, followed by Northeast. Municipalities with low and very low MHDI, with populational support up to 25,000 inhabitants, and those that did not have centralized purchases showed the highest values of ER for researched medicines in the years studied.

Appendix 2 shows all the ER values and average prices paid for medicines, according to all analyses.

## DISCUSSION

The study reveals worrying trends on medicines prices in public municipal purchases for primary attention. Municipalities with low and very low MHDI, such as the smallest ones, with up to 25,000 inhabitants, and that do not buy in associations constantly show higher prices. The highest prices in these municipalities may be because of limited economies, low supplier offers, logistic barriers, lower negotiation power, and fragilized local management.

These results show the urgent need for intervention regarding normative, market, and management aspects to supply the access of essential medicines to the population since the prizes affect directly the acquisition by the municipalities. In the presence of incompatible variations, it is crucial to promote regulation, intervention, or negotiation strategies to reduce financial barriers, ensuring efficient public management and the population’s right to have access to medicines.

Inequality on the prices of public purchase practices was also demonstrated to the *Componente Especializado da Assistência Farmacêutica* (CEAF – Specialized Compound of Pharmaceutical Services), highlighting a trend on lower prices for the most populous and economically favored federal unities^2^. The broad variation in prices indicates challenges on acquisition, elevating costs for federal unities with lower negotiation power. This aggravates disparities in the access to medicines on SUS, especially in socioeconomically vulnerable regions and with low demand, where the prices tend to be higher^20^.

The North region showed higher average prices in the three years analyzed. On the Northeast, the values also surpassed the averages of Midwest, South, and Southeast, evidencing regional inequalities on medicines acquisition.

Along with other studies about financing and structuring of municipal pharmaceutical services, the results indicate a complex scenario of vulnerabilities in management and services, impacting the capacity of many Brazilian municipalities in guaranteeing access to medicines in PHC.

Silva et al.^10^ (2024) observed that, in 2019, the municipalities sampled in their survey spent, on average, R$ 45.09 on medicines purchased for their citizens, an amount well above the sum of the CBAF counterparts, which could reach a maximum, at that time, of only R$ 10.00, an amount expected in regulations for municipalities with the lowest MHDI. In the same study, they exposed the inequality of investments in medicines, with municipalities with higher vulnerability being able to invest only R$ 30.00 on average. Between the regions, the disparities were also clear, with the municipalities in the North and Northeast regions showing the lowest investments in medicines that year: R$ 23.58 and R$ 24.20, respectively. When looking at the per capita investment data in medicines in the municipalities, from 2010 to 2019, note that in the regions where there was an increase in the value (Midwest, Southeast, and South), this was very small. Nevertheless, the Northeast and North regions, which invested less than the national average throughout the period, showed a decrease in the value of investment, during the period surveyed, of 25.63% and 21.69%^10^.

In addition to purchasing medicines with the highest prices and having the lowest per capita investments in medicines, there are also conditions already described in the literature of lower infrastructure development, qualified workforce and organization of the management of pharmaceutical services in the North and Northeast regions compared to the other regions^5,21,22^. The impact of this combination can be seen in the results of the PNAUM: the general availability of medicines in primary health care units varied according to the regions of the country, which is lower in the North and Northeast regions (44.6% and 46.3%, respectively)^5^ and total access to medicines by the population is also lower in these regions^9^.

The tax on the circulation of goods and services, responsible for most of the tax burden in the field of medicines, which varies from 12% for states such as Minas Gerais and São Paulo, to 21% for states such as Piauí, also contributes to the inequalities in prices^23^. In Brazil, the maximum sale price to the government, the ceiling price that must be observed in purchases destined for the SUS, is the result of the application of the price adequacy coefficient (CAP – coeficiente de adequação de preços) on the factory price. The CAP, regulated by Resolution No. 3, of March 2^nd^, 2011, is a mandatory minimum discount to be applied whenever sales of medicines included in the list attached to Communiqué No. 15, of August 31, 2017 – Consolidated Version, are made^24^.

The North region is especially concerning due to its natural and social conditions that strongly impact on the cost of transporting products to their municipalities and storing them. The so-called “Amazon factor”^25^, which includes the impressive distances between the municipalities, the dependence on river transport, which is subject to climatic conditions for its operation, the low population densities of the municipalities, among other economic and contextual factors, place the municipalities in the region in difficult conditions for the acquisition of medicines that cannot be compared with other places. The North region also has the health regions with the worst performance in topics such as maternal and reproductive health and healthcare system coverage, in addition to being the region of the country with the worst evaluations of PHC services^26^. However, the transfer values for the acquisition of PHC medicines do not consider these regional conditions, and they are made available to municipalities in the other regions.

It is important to consider the way in which municipalities act in their contexts since health expenditures are not immune to diseconomies of scale — related to the population size of the municipalities and health responsibilities. Economic aspects, such as the scale of the acquisition of medicines and negotiating power, can explain the differences in spending between the federated entities, especially the municipalities. As an example, one can mention the purchase of medicines made in local pharmacies/drugstores (58.3%), particularly in the municipalities of the North region (73.4%), which indicates inadequacies in the application of public resources in the SUS^27^. However, empty bids and other barriers in the daily life of public management need to be considered and faced more forcefully.

The associated purchase of medicines, especially in intermunicipal consortia or with the state government, proved to be an important strategy to reduce the prices in general. This pattern was observed in all years and for all medicines, suggesting an efficient way to circumvent the barriers imposed by the contextual conditions of smaller municipalities for public procurement.

The positive experience in the public purchase of medicines in consortia has been reported for some years. In the Cariri region, in Ceará, for example, the acquisitions made by the Health Consortia between 2017 and 2018 showed lower unit values for most items than the municipal acquisitions. That is 19 times for the average value, 18 times for the weighted average value, and 20 times for the median value^28^. The purchase in consortium gains in volume, attracting greater interest from suppliers, and there is a reduction in costs due to greater negotiating power.

The execution of larger purchase contracts with larger orders, fixed terms, and staggered deliveries have advantages such as regularity in supply, reduction of inventories and storage costs, guarantee of medicines with favorable expiration dates and planned and gradual financial execution^27^.

The difficulties in obtaining medicines in PHC result in the difficulty of free access to medicines, especially those of continuous use, which can compromise the family budget and favor the abandonment of treatment, with worsening of the health condition and the consequent increase in spending on outpatient care and hospitalizations. Individuals with lower income and low level of education had a greater chance of access to medicines via SUS (from 1998 to 2008), which highlights the importance of the SUS for the promotion of equity in access to medicines^29^.

According to the *Pesquisa Nacional por Amostra de Domicílios* (PNAD – National Household Sample Survey) conducted in Brazil in 1998, 2003, and 2008, the evolution of medicines provision costs has contributed to the increase in free access to medicines by the SUS, but it was insufficient to reduce the inequality in their access in the period analyzed. The trajectory of underfunding of the SUS and the imbalance between public and private spending on medicines are among the barriers to the implementation of policies, which may have contributed to the maintenance of inequalities^30^.

One limitation of this research is the use of secondary data in the comparisons between the prices charged in the purchase of medicines by the municipalities, as they are self-declared data obtained by an information system that, although official and regulated, does not have the obligation and adherence of 100% of the municipalities or the completion of the information of 100% of the purchases made. The measurement of the total quantities acquired by each municipality per year is also not possible since the data record is not sufficient for such a conclusion. However, the study has the largest amount of data observed for this theme in literature, much higher than the information from the Health Price Database.

This study, of an innovative nature, analyzes the prices practiced in the acquisition of medicines by Brazilian municipalities based on BNAFAR data and suggests an indicator that enables a comparative evaluation of the general averages of prices paid, can collaborate to improve the management processes of pharmaceutical services, favoring the negotiations of municipalities in their acquisition of medicines, fostering the transparency and accountability.

## CONCLUSION

This study highlights the serious inequality in the prices of medicines purchased by Brazilian municipalities. It is urgent to implement strategies to avoid increasing the disparity in the supply of medicines between municipalities, especially the smallest and most vulnerable, regionally or in relation to MHDI, as identified by the averages of the ER indicator. This can reduce costs and improve quality of life, resulting in a more efficient application of public resources.

The ER indicator, adapted from an index proposed for international analyses by the WHO, proved to be useful for comparing price patterns paid by municipalities in the acquisition of medicines, and it was possible to reveal that municipalities with social and economic characteristics of greater vulnerability are the most affected by the prices paid for medicines. Considering the scarcity of resources, it is essential to advance in the evaluation of efficiency and investments in medicines by local governments, especially in their availability in Brazilian municipalities.

## Supplementary material

Apendix 1 available from: https://figshare.com/s/a8e51658714a2ad406ab

Apendix 2 available from: https://figshare.com/s/834607e5f76d9ddbe649

## Data Availability

The study data are available on a digital platform (links in the article text) or upon request to the authors.
