# Supplementary material for: Prices paid for primary health care medicines by Brazilian municipalities
Source: Rev Saude Publica. 2025 Dec 8;59:e45. doi: 10.11606/s1518-8787.2025059006964 (PMC12696822; doi:10.11606/s1518-8787.2025059006964)
Supplement: Translation

# Translation: Preços pagos pelos medicamentos para atenção primária pelos municípios brasileiros

**Authors:** Wendell Rodrigues Oliveira da Silva, Ivanessa Thaiane do Nascimento Cavalcanti, José Roberto Peters, Luis Eduardo Maciel dos Santos Ferreira, Rafael Santos Santana, Silvana Nair Leite

**Affiliations:** I Universidade de Brasília. Faculdade de Ciências da Saúde, Programa de Pós-Graduação em Ciências Farmacêuticas. Brasília, DF, Brasil; II Ministério da Saúde. Departamento de Economia e Desenvolvimento em Saúde. Brasília, DF, Brasil; III Universidade Federal de Santa Catarina. Departamento de Ciências Farmacêuticas. Programa de Pós-Graduação em Assistência Farmacêutica. Florianópolis, SC, Brasil

**Keywords:** Assistência Farmacêutica, Atenção Primária à Saúde, Custos de Medicamentos, Equidade na Alocação de Recursos, Política Nacional de Assistência Farmacêutica

## Abstract

**OBJETIVO:**

Analisar os preços pagos por medicamentos entre os municípios brasileiros em 2016, 2018 e 2020, comparando índice de desenvolvimento humano municipal, porte, região e modalidade de compra.

**MÉTODOS:**

O estudo utiliza o indicador Razão Econômica, adaptado a partir do proposto para análises internacionais pela Organização Mundial da Saúde, e faz análises com os dados alimentados pelos municípios na Base Nacional de Dados de Ações e Serviços da Assistência Farmacêutica.

**RESULTADOS:**

O estudo revela que os municípios social e economicamente mais vulneráveis pagam mais caro por esses medicamentos, especialmente nas regiões Norte e Nordeste, o que pode comprometer o acesso e a eficiência do Sistema Único de Saúde. Além disso, o artigo demonstra que municípios que realizam compras em associação tendem a pagar preços menores, sugerindo que consórcios podem ser uma estratégia para mitigar as desigualdades nos preços.

**CONCLUSÕES:**

Com os resultados obtidos, propõe-se a adoção de estratégias regulatórias, econômicas e de negociação para reduzir essas diferenças e garantir uma distribuição mais equitativa dos medicamentos no Brasil.

## INTRODUÇÃO

Os altos preços dos medicamentos preocupam governos, formuladores de políticas, seguradores e pacientes, pois comprometem seu acesso equitativo e ameaçam a sustentabilidade dos sistemas de saúde, independentemente do grau de desenvolvimento socioeconômico do país^1^.

Os preços dos medicamentos oscilam segundo as interações entre oferta e demanda, dentro do escopo de regulamentação dos preços do país^1^. Entre os países, fatores como o sistema de saúde, a política de medicamentos e o acesso gratuito influenciam o preço e, consequentemente, a obtenção dos medicamentos^4,5^. No Brasil, estudos sobre preço, acesso e qualidade dos serviços farmacêuticos são essenciais, já que o fornecimento público de medicamentos é a principal forma de acesso para a população dependente do sistema público de saúde^5^.

Vigorando há mais de 20 anos, a Lei nº 10.742/2003 estabelece teto de preços para medicamentos no Brasil. Mas, na realidade do mercado farmacêutico, os preços podem variar mais de 2.500% no mercado privado^3^. Relatório do Tribunal de Contas da União identificou preços registrados na tabela da Câmara de Regulação do Mercado de Medicamentos (CMED) acima de 10.000% ao praticado em compras públicas^6^.

**Tabela:** Comparação dos valores médios gerais de razão econômica nos anos analisados, IDHM, tipo de compra, região do país e porte populacionala.

	Média (IC95%)
Região	
Centro-Oeste	1,04 (1,00–1,07)
Nordeste	1,13 (1,10–1,16)
Norte	1,32 (1,28–1,37)
Sudeste	0,82 (0,79–0,85)
Sul	0,78 (0,74–8,82)
Porte populacional	
Até 25.000 hab.	1,02 (1,01–1,03)
25.001 a 50.000 hab.	0,98 (0,97–1,00)
50.001 a 100.000 hab.	0,93 (0,91–0,95)
100.000 a 500.000 hab.	0,93 (0,91–0,95)
Acima de 500.000 hab.	0,88 (0,84–0,92)
IDHM	
Muito baixo	1,37 (1,26–1,47)
Baixo	1,18 (1,16–1,20)
Médio	0,97 (0,96–0,99)
Alto	0,85 (0,82–0,87)
Muito alto	0,75 (0,69–0,80)
Compras associadas	
Compra associada	0,82 (0,78–0,85)
Compra sem associação	1,10 (1,08–1,12)

IDHM: Índice de Desenvolvimento Humano Municipal; IC95%: intervalo de confiança de 95%.

^a^ Nesta análise, os valores encontrados para o *P*-valor foram menores que 0,0001, mostrando que os resultados são estatisticamente significantes para descrever o efeito do modelo.

A oferta de medicamentos depende do financiamento da saúde, gestão eficiente, disponibilidade de produtos, poder aquisitivo, racionalidade do uso e acessibilidade geográfica^7^. O financiamento público e a organização da assistência farmacêutica na atenção primária desempenham papel fundamental no acesso aos medicamentos pela população brasileira^5,8,9^. Em 2015, 47% da população obtinha todos os seus medicamentos para doenças crônicas de forma gratuita no Sistema Único de Saúde (SUS). No entanto, a região Nordeste apresentou a menor prevalência de acesso a todos os medicamentos^8^.

No SUS, as compras de medicamentos são realizadas de forma autônoma pela União, os estados, o Distrito Federal e os municípios, com descentralização que permite planejamento conforme necessidades locais. A maioria dos medicamentos da atenção primária é adquirida diretamente pelos municípios, de forma isolada ou em consórcios. Em 2019, a União investiu R$ 132 bilhões em saúde e R$ 13 bilhões em medicamentos, enquanto estados gastaram R$ 102 bilhões e R$ 1,7 bilhão, respectivamente. Municípios investiram R$ 5 bilhões em medicamentos, segundo dados do Sistema de Informações sobre Orçamentos Públicos em Saúde (SIOPS)^10^.

A Portaria nº 3.193/2019 alterou os repasses do Componente Básico da Assistência Farmacêutica (CBAF), vinculando-os ao índice de desenvolvimento humano municipal (IDHM) dos municípios. A União repassava valores *per capita* aos municípios anualmente, variando conforme a classificação do IDHM, dados que foram atualizados em 2025^11^. O financiamento visa a aquisição de medicamentos e insumos incluídos na Relação Nacional de Medicamentos Essenciais (Rename) para a Atenção Primária no SUS.

Apesar da descentralização, o financiamento municipal para medicamentos é preocupante: municípios mais vulneráveis investem menos e dependem mais dos repasses federais, segundo Silva et al.^10^ (2024), tendência observada entre 2016 e 2020.

Considerando as condições econômicas e de gestão municipal e as características do mercado farmacêutico no Brasil, os preços dos medicamentos para atenção primária podem variar bastante entre os municípios^12^ e representar um importante impacto na capacidade municipal para garantir a disponibilidade destes medicamentos, essenciais para a resolubilidade da atenção primária e a obtenção de melhores resultados em saúde.

O presente estudo analisou os preços praticados em 2016, 2018 e 2020 na aquisição, pelos municípios brasileiros, de medicamentos utilizados na atenção primária, considerando suas condições socioeconômicas, região geográfica e modalidades de aquisição.

## MÉTODOS

Trata-se de um estudo exploratório, retrospectivo, no qual se analisou os preços das aquisições de medicamentos da atenção primária pelos municípios, a partir dos dados alimentados na Base Nacional de Dados e Ações e Serviços da Assistência Farmacêutica (BNAFAR). Ela integra e consolida os dados nacionais de posição de estoque, entradas, saídas e dispensações realizadas pelos estabelecimentos de saúde para os medicamentos da Rename e do Programa Farmácia Popular do Brasil, contemplando o maior número de registros de compras de medicamentos disponíveis, especialmente de municípios^13^.

Para determinar o valor pago pelos medicamentos, extraiu-se os valores registrados, tendo como referência as apresentações mais utilizadas dos medicamentos, sem discriminação de marcas. Os dados foram referentes a 2016 (2.440 municípios), 2018 (2.866 municípios) e 2020 (3.815 municípios). A escolha desses anos considerou as correções do valor da contrapartida do CBAF por parte do Ministério da Saúde em 2017^14^ e em 2019^11^. Os dados foram coletados utilizando *Business Intelligence* (BI) da MicroStrategy Office*.*

A escolha dos medicamentos se baseou nos identificados na Pesquisa Nacional sobre Acesso, Utilização e Promoção do Uso Racional de Medicamentos (PNAUM) como os mais utilizados para hipertensão arterial, diabetes mellitus, hipercolesterolemia, doença respiratória pulmonar crônica, depressão, reumatismo, dor, febre e infecção. Para os três últimos, como a PNAUM não encontrou diferença de utilização, os autores decidiram por medicamentos de amplo uso pela população brasileira^15^.

Os medicamentos selecionados para análise estão descritos no Apêndice 1^a^. Utilizou-se o programa estatístico Rstudio para exclusão dos *outliers* do banco de dados, considerando a percentagem abaixo de 1% e acima de 99% como pontos fora da curva.

Os dados da BNAFAR foram extraídos das apresentações adquiridas pelos municípios, com quantidades e preços praticados, e calculou-se a média dos preços pagos por unidade de fornecimento.

Realizou-se a atualização monetária dos valores pagos pelos medicamentos. Esses valores foram deflacionados para dezembro/2022, de acordo com a variação anual do índice nacional de preços ao consumidor amplo (IPCA), calculado pelo Instituto Brasileiro de Geografia e Estatística – IBGE (2016: 44,32%; 2018: 31,54%; 2020: 21,88%). A escolha desse deflator baseou-se na Lei nº 10.742/2003, que estabeleceu as normas de regulação do setor farmacêutico e definiu o índice para fins de ajuste nos preços dos medicamentos no país.

A forma de compra dos medicamentos pelos municípios foi classificada entre compra em associação ou não associada, distinguindo aquelas em conjunto entre os entes (município e estado), como consórcios intermunicipais, em âmbito regional ou estadual, e nas situações em que o município adquire seus medicamentos isoladamente. Esse dado foi fornecido pela Coordenação-Geral da Assistência Farmacêutica Básica do Ministério da Saúde.

Quanto ao porte populacional municipal, utilizou-se o modelo de estrato do Programa Qualifar-SUS, que estratifica os municípios em: até 25.000 habitantes, de 25.001 a 50.000 habitantes, de 50.001 a 100.000 habitantes, de 100.001 a 500.000 habitantes e municípios acima de 500.000 habitantes^16^.

Utilizou-se também o índice de desenvolvimento humano municipal (IDHM). Este varia entre 0 e 1 (PNUD), sendo considerado IDHM muito baixo os municípios com índice abaixo de 0,499; IDHM baixo os municípios com índice entre 0,500 e 0,599; IDHM médio os municípios com índice entre 0,600 e 0,699; IDHM alto os municípios com índice entre 0,700 e 0,799; e IDHM muito alto os municípios com índice igual ou superior a 0,800.

A partir desses dados, realizaram-se análises regionais, por porte populacional, por estrato de IDHM e por realização de compras em associação ou não, para identificar e comparar os valores praticados na compra de medicamentos pelos municípios brasileiros.

Utilizou-se uma adaptação da “razão de preço de medicamento”, índice desenvolvido pela Organização Mundial da Saúde (OMS)/Ação de Saúde Internacional^17^. Ele é uma ferramenta metodológica para medir os preços dos medicamentos (preços de compras governamentais e preços aos pacientes), a disponibilidade, a acessibilidade e os componentes do preço, elaborada anteriormente pela OMS^18,19^.

Neste estudo, adaptou-se o indicador para contemplar as informações processadas na amostra analisada e comparar os valores pagos, considerando os diferentes medicamentos incluídos na amostra. Estabeleceu-se como “preço unitário médio local” a média do preço médio pago para cada apresentação dos medicamentos por município, e para “preço unitário de referência nacional” o preço médio nacional pago pela apresentação dos medicamentos que corresponde à média nacional dos valores pagos para cada apresentação de cada medicamento, considerando todos os registros para a compra de tal medicamento na BNAFAR, naquele ano. Assim, a fórmula da Razão Econômica (RE) adaptada utilizada neste estudo foi:


$$\mathrm{RE}=\frac{\begin{array}{c} \text{ média do preço médio pago por apresentação }\\ \text{ dos medicamentos pelo município } \end{array}}{\text{ preço médio nacional da apresentação dos medicamentos }}$$

Como resultado do indicador, os valores obtidos para a RE igual a 1 sinalizam que o valor pago foi idêntico à média nacional; para um resultado menor que 1, indica que o preço pago foi menor que o valor pago para a aquisição do medicamento quando comparado à média nacional; e para um resultado maior que 1, mostra que houve um pagamento acima da média nacional.

O modelo de equações de estimações generalizadas foi usado para comparar a média da variável RE entre as variáveis estudadas. O nível de significância adotado foi 0,05. As análises foram realizadas no software IBM SPSS Statistics v.25.

## RESULTADOS

Os municípios deste estudo caracterizam-se da seguinte forma: em relação à região geográfica, 351 são da região Centro-Oeste, 1.147 são do Nordeste, 256 são do Norte, 1.310 são do Sudeste e 751 são da região Sul. Quanto ao porte populacional: 2.894 possuem até 25.000 habitantes, 476 possuem de 25.001 a 50.000 habitantes, 233 possuem de 50.001 a 100.000 habitantes, 181 possuem de 100.000 a 500.000 habitantes e 31 municípios possuem acima de 500.000 habitantes. No que concerne à classificação do IDHM, a distribuição apresenta-se da seguinte forma: 10 municípios são classificados como muito baixo, 847 baixo, 1.680 médio, 1.250 alto e 28 como IDHM muito alto. E referente à estratégia de aquisição (com ou sem associação), 1.375 municípios realizam compras associadas, 2.391 realizam compras sem associação com outros entes e de 49 municípios não se conseguiu obter essa informação.

Os resultados obtidos para preços praticados nas aquisições por medicamento e os RE correspondentes por região, porte do município, faixa de IDHM e modalidade de aquisição são apresentados detalhadamente no material complementar Apêndice 2^b^. Na Figura 1, é apresentado o valor médio da RE dos medicamentos, regionalmente, por ano. É possível observar que nos três anos da pesquisa a região Norte apresentou a maior média do valor da RE.

**Figura 1:** Razão econômica média dos medicamentos analisados por região e por ano.

Considerando o porte populacional (Figura 2), pode-se observar que em 2016, 2018 e 2020, os municípios menores, com até 25.000 hab., apresentaram a maior média do valor de RE.

**Figura 2:** Razão econômica média dos medicamentos analisados por porte populacional e por ano.

Na Figura 3, que analisa o valor médio da RE dos medicamentos por estrato de IDHM, nota-se que, nos anos pesquisados, os municípios com IDHM muito baixo apresentaram a maior média do valor de RE.

**Figura 3:** Razão econômica média dos medicamentos analisados por IDHM e por ano.

IDHM: Índice de Desenvolvimento Humano Municipal.

Ao analisar as compras de forma associada ou não (Figura 4), tem-se que o valor médio da RE em 2016, 2018 e 2020 nos municípios que não realizaram a compra em associação foi maior em todos os anos pesquisados.

**Figura 4:** Razão econômica média dos medicamentos analisados por compra associada e sem associação, por ano.

A Tabela apresenta a comparação dos valores médios de RE dentre todos os medicamentos, nas variáveis estudadas. A região Norte tem a maior média geral de valores de RE, seguida pelo Nordeste. Os municípios de IDHM muito baixo e baixo, com o porte populacional de até 25.000 habitantes, e aqueles que não fizeram compras centralizadas apresentaram os maiores valores de RE para os medicamentos pesquisados, nos anos estudados.

Todos os valores de RE e dos preços médios pagos por medicamentos, de acordo com todas as análises realizadas, encontram-se no Apêndice 2.

## DISCUSSÃO

O estudo revela tendências preocupantes nos preços de medicamentos em compras públicas municipais para a atenção primária. Municípios com IDHM baixo e muito baixo, assim como os menores, com até 25.000 habitantes, e que não realizam as compras em associações apresentam consistentemente preços mais altos. Os maiores preços nesses municípios podem decorrer de economias de escala limitada, baixa oferta de fornecedores, barreiras logísticas, menor poder de negociação e gestão local fragilizada.

Esses resultados evidenciam a necessidade urgente de intervenções normativas, de mercado e de gestão para garantir o acesso a medicamentos essenciais para a população, já que o preço afeta diretamente a obtenção pelos municípios. Diante de variações incompatíveis, é crucial promover estratégias de regulação, intervenção ou negociação para reduzir barreiras financeiras, garantindo uma gestão pública eficiente e o direito da população aos medicamentos^19^.

A desigualdade nos preços praticados nas compras públicas também já foi demonstrada para o Componente Especializado da Assistência Farmacêutica (Ceaf), destacando uma tendência de preços mais baixos para as unidades federativas mais populosas e economicamente favorecidas^2^. A ampla variação nos preços indica desafios na aquisição, elevando custos para unidades federativas com menor poder de negociação. Isso agrava disparidades no acesso a medicamentos no SUS, especialmente em regiões socioeconomicamente vulneráveis e com baixa demanda, onde os preços tendem a ser maiores^20^.

A região Norte apresentou preços médios mais altos nos três anos analisados. No Nordeste, os valores também superaram as médias do Centro-Oeste, Sul e Sudeste, evidenciando desigualdades regionais na aquisição de medicamentos.

Os resultados, somados a outros estudos sobre financiamento e estruturação da assistência farmacêutica municipal, indicam um cenário complexo de vulnerabilidades na gestão e nos serviços, impactando a capacidade de muitos municípios brasileiros em garantir o acesso a medicamentos na atenção primária.

Silva et al. (2024)^10^ observaram que, em 2019, os municípios amostrados em sua pesquisa gastaram, em média, R$ 45,09 com medicamentos adquiridos para seus munícipes, um valor bem acima da soma das contrapartidas do CBAF, que podiam chegar no máximo, nessa época, a apenas R$ 10,00, valor previsto em normativa para os municípios com os menores IDHM. No mesmo estudo, denunciam a desigualdade de investimentos em medicamentos, tendo os municípios com vulnerabilidade mais alta podendo investir apenas R$30,00 em média. Entre as regiões, as disparidades também foram claras, com os municípios das regiões Norte e Nordeste com os menores investimentos em medicamentos naquele ano: R$ 23,58 e R$ 24,20, respectivamente. Já quando se observa os dados de investimento *per capita* em medicamentos dos municípios, de 2010 a 2019, é possível observar que nas regiões em que houve aumento do valor (Centro-Oeste, Sudeste e Sul), esse foi muito pequeno. Já as regiões Nordeste e Norte, que em todo o período investiram menos que a média nacional, apresentaram recuo no valor do investimento, durante o intervalo pesquisado, de 25,63% e 21,69%^10^.

A prática de aquisições com preços mais elevados e os menores investimentos *per capita* em medicamentos se somam, ainda, a condições já descritas na literatura de menor desenvolvimento de infraestrutura, força de trabalho qualificada e organização da gestão da assistência farmacêutica nas regiões Norte e Nordeste em comparação com as demais regiões^5,21,22^. O impacto de tal combinação pode ser visualizado nos resultados da PNAUM: a disponibilidade geral de medicamentos nas unidades básicas de saúde variou conforme as regiões do país, sendo inferior nas regiões Norte e Nordeste (44,6% e 46,3%, respectivamente)^5^ e o acesso total aos medicamentos pela população também é menor nessas regiões^9^.

Contribui para as desigualdades de preços praticados, também, o imposto sobre circulação de mercadorias e prestação de serviços (ICMS), responsável pela maior parte da carga tributária no âmbito dos medicamentos, que entre os estados varia de 12%, para estados como Minas Gerais e São Paulo, a 21% para estados como o Piauí^23^. No Brasil, o preço máximo de venda ao governo (PMVG), preço-teto que deve ser observado nas aquisições destinadas ao SUS, é o resultado da aplicação do coeficiente de adequação de preços (CAP) sobre o preço fábrica. O CAP, regulamentado pela Resolução nº 3, de 2 de março de 2011, é um desconto mínimo obrigatório a ser aplicado sempre que forem realizadas vendas de medicamentos constantes do rol anexo ao Comunicado nº 15, de 31 de agosto de 2017 - Versão Consolidada, ou para atender ordem judicial^24^.

A região Norte causa especial preocupação por suas condições naturais e sociais que impactam fortemente o custo para o transporte dos produtos até seus municípios e o armazenamento. O chamado “fator amazônico”^25^, que inclui as impressionantes distâncias entre os municípios, a dependência de transporte fluvial, que está sujeito às condições climáticas para seu funcionamento, as baixas densidades populacionais dos municípios, entre outros fatores econômicos e contextuais colocam os municípios da região em condições de dificuldades para a aquisição de medicamentos que não podem ser comparáveis com os demais locais. A região Norte também é a que concentra as regiões de saúde com pior desempenho em temas como saúde materna e reprodutiva e cobertura do sistema de saúde, além de ser a região do país com as piores avaliações dos serviços da atenção primária^26^. No entanto, os valores de repasse para a aquisição de medicamentos da atenção primária não consideram estas condições regionais, sendo os mesmos disponibilizados para os municípios das demais regiões.

É importante ponderar a forma como os municípios atuam em seus contextos, pois os gastos em saúde não estão imunes a deseconomias de escala, relacionadas ao porte populacional dos municípios e responsabilidades sanitárias, pois aspectos econômicos, tais como escala da aquisição de medicamentos e poder de negociação, podem explicar as diferenças de gasto entre os entes federados, especialmente os municípios. Como exemplo, pode-se citar a compra de medicamentos realizada em farmácias/drogarias locais (58,3%), particularmente nos municípios da região Norte (73,4%), o que sinaliza para inadequações na aplicação de recursos públicos no SUS^27^. No entanto, licitações vazias e outras barreiras no cotidiano da gestão pública precisam ser consideradas e enfrentadas mais contundentemente.

A compra associada de medicamentos, principalmente em consórcios intermunicipais ou com governo estadual, se mostrou uma estratégia importante para reduzir os preços pagos em geral. Esse padrão foi observado em todos os anos e para todos os medicamentos, sugerindo uma via eficiente para driblar as barreiras impostas pelas condições contextuais dos municípios menores para as compras públicas.

A experiência positiva na compra pública de medicamentos em consórcios tem sido relatada há alguns anos. Na região do Cariri, no Ceará, por exemplo, as aquisições realizadas pelos Consórcios de Saúde entre 2017 e 2018 apresentaram valores unitários menores para a maioria dos itens do que as aquisições municipais. São 19 vezes para o valor médio, 18 vezes para o valor médio ponderado e 20 vezes para o valor mediano^28^. A compra em consórcio ganha em volume, atraindo maior interesse dos fornecedores, e há uma redução de custos devido ao maior poder de negociação.

A realização de contratos de compra com volume maior, prazo determinado e entregas parceladas apresenta vantagens como regularidade no abastecimento, redução dos estoques e nos custos de armazenamento, garantia de medicamentos com prazos de validade favoráveis e execução financeira planejada e gradual^27^.

As dificuldades para a obtenção de medicamentos na atenção primária têm como consequência a dificuldade de acesso gratuito aos medicamentos, especialmente os de uso contínuo, que pode causar o comprometimento do orçamento familiar ou favorecer o abandono do tratamento, com agravo do estado de saúde e o consequente aumento dos gastos em atendimentos ambulatoriais e internações. Os indivíduos de menor renda e baixo nível de escolaridade tiveram maior chance de acesso a medicamentos pelo SUS (entre 1998 e 2008), o que demonstra a importância do SUS para a promoção da equidade no acesso a medicamentos, e como tal precisa ser valorizado^29^.

Segundo a Pesquisa Nacional por Amostra de Domicílios (PNAD) realizada no Brasil em 1998, 2003 e 2008, a evolução dos gastos na provisão de medicamentos tem contribuído para a elevação do acesso gratuito a medicamentos pelo SUS, mas foi insuficiente para reduzir, no período analisado, a desigualdade no seu acesso. A trajetória de subfinanciamento do SUS e o desequilíbrio entre o gasto público e o privado com medicamentos estão entre as barreiras para a efetivação das políticas, o que pode ter colaborado para a manutenção das desigualdades^30^.

Pode-se citar como uma limitação desta pesquisa a utilização de dados secundários nas comparações entre os preços praticados na compra de medicamentos pelos municípios, pois são dados autodeclarados e obtidos por um sistema de informação que, ainda que seja oficial e regulado, não conta com a obrigatoriedade e adesão de 100% dos municípios ou o preenchimento das informações de 100% das compras realizadas. Também não é possível aferir as quantidades totais adquiridas por cada município por ano, uma vez que o registro de dados não se mostra suficiente para tal conclusão. No entanto, o estudo apresenta o maior número de dados observados para esta temática já apresentado na literatura, muito superior às informações oriundas do Banco de Preços em Saúde.

Este estudo, de caráter inovador, por analisar os preços praticados na aquisição de medicamentos pelos municípios brasileiros a partir de dados da BNAFAR e por sugerir um indicador que permite avaliar comparativamente as médias gerais de preços pagos, pode colaborar para a melhoria dos processos de gestão da assistência farmacêutica, favorecendo as negociações dos municípios nas suas aquisições de medicamentos, fomentando a transparência e prestação de contas.

## CONCLUSÃO

Este estudo destaca a grave desigualdade nos preços dos medicamentos adquiridos pelos municípios brasileiros. É urgente implementar estratégias para evitar o aumento da disparidade na oferta de medicamentos entre os municípios, especialmente os menores e os mais vulneráveis, regionalmente ou em relação ao IDHM, conforme identificado pelas médias dos valores do indicador Razão Econômica (RE). Isso pode reduzir os custos e melhorar a qualidade de vida, resultando em uma aplicação mais eficiente dos recursos públicos.

O indicador RE, adaptado a partir de índice proposto para análises internacionais da OMS, mostrou-se útil para comparar padrões de preços pagos por municípios na aquisição de medicamentos, sendo possível revelar que municípios com características sociais e econômicas de maior vulnerabilidade são os mais prejudicados pelos preços pagos nos medicamentos. Considerando a escassez de recursos, é fundamental avançar na avaliação da eficiência e dos investimentos em medicamentos pelos governos locais, especialmente na sua disponibilização nos municípios brasileiros.

## Material suplementar

Apêndice 1 disponível em: https://figshare.com/s/834607e5f76d9ddbe649

Apêndice 2 disponível em: https://figshare.com/s/a8e51658714a2ad406ab

## Data Availability

Os dados do estudo estão disponibilizados em plataforma digital (links no texto do artigo) ou mediante solicitação aos autores.
